# Visualizing interferential stimulation of human brains

**DOI:** 10.3389/fnhum.2023.1239114

**Published:** 2023-10-26

**Authors:** Yu Huang

**Affiliations:** Research and Development, Soterix Medical Inc., Woodbridge, NJ, United States

**Keywords:** interferential stimulation, temporal interference, visualization, computational modeling, transcranial electric stimulation

## Abstract

**Introduction:**

Transcranial electrical stimulation (TES) is limited in focally stimulating deep-brain regions, even with optimized stimulation montages. Recently, interferential stimulation (IFS), also known as transcranial temporal interference stimulation (TI, TIS, or tTIS), has drawn much attention in the TES community as both computational and experimental studies show that IFS can reach deep-brain areas. However, the underlying electrodynamics of IFS is complicated and difficult to visualize. Existing literature only shows static visualization of the interfered electric field induced by IFS. These could result in a simplified understanding that there is always one static focal spot between the two pairs of stimulation electrodes. This static visualization can be frequently found in the IFS literature. Here, we aimed to systematically visualize the entire dynamics of IFS.

**Methods and results:**

Following the previous study, the lead field was solved for the MNI-152 head, and optimal montages using either two pairs of electrodes or two arrays of electrodes were found to stimulate a deep-brain region close to the left striatum with the highest possible focality. We then visualized the two stimulating electrical currents injected with similar frequencies. We animated the instant electric field vector at the target and one exemplary off-target location both in 3D space and as a 2D Lissajous curve. We finally visualized the distribution of the interfered electric field and the amplitude modulation envelope at an axial slice going through the target location. These two quantities were visualized in two directions: radial-in and posterior–anterior.

**Discussion:**

We hope that with intuitive visualization, this study can contribute as an educational resource to the community’s understanding of IFS as a powerful modality for non-invasive focal deep-brain stimulation.

## Introduction

**FIGURE 1:**
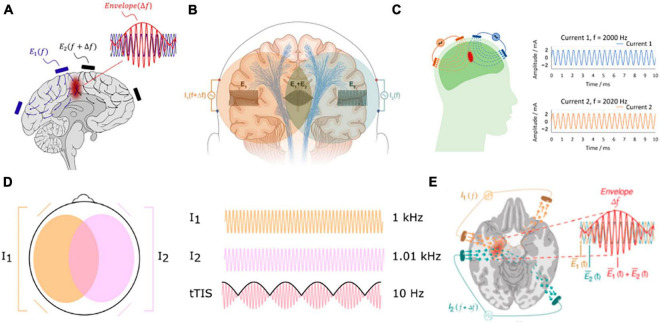
Compilation of commonly used visualization of IFS in the literature. **(A)** Grossman et al. (2017); **(B)** Mirzakhalili et al. (2020); **(C)** Piao et al. (2022); **(D)** von Conta et al. (2021); **(E)** Violante et al. (2022).

As a non-invasive brain stimulation method, transcranial electrical stimulation (TES) has been shown to improve cognitive functions and help treat some neurological diseases such as major depression (Bikson et al., 2008), epilepsy (Fregni et al., 2006b; Auvichayapat et al., 2013), Parkinson’s disease (Fregni et al., 2006a), chronic pain (Fregni et al., 2007), and stroke (Meinzer et al., 2016). However, TES is not able to focally stimulate deep-brain regions, even with optimized stimulation montages (Dmochowski et al., 2011; Huang and Parra, 2019). Recently, interferential stimulation (IFS), also known as transcranial temporal interference stimulation (TI, TIS, or tTIS), has drawn much attention in the TES community as both computational and experimental studies show that IFS can reach deep-brain areas (Grossman et al., 2017; Huang et al., 2020; Huang and Datta, 2021; Violante et al., 2022). When optimized, it can achieve higher focality than conventional TES (Huang et al., 2020). However, the underlying electrodynamics of IFS is complicated and difficult to visualize. This is because the interfered electric field is amplitude modulated and contains both a fast-oscillating carrier signal in the kilohertz range and a slowly oscillating modulation envelope in ∼10 Hz. The premise of IFS is that neurons only respond to slower oscillation due to their property of low-pass filtering (Grossman et al., 2017). To the best of our knowledge, except for a conference poster that acknowledges the rotational property of the interfered electric field (Turovets et al., 2018), existing literature only shows static visualization of the interfered electric field induced by IFS (Grossman et al., 2017; Rampersad et al., 2019; Lee et al., 2020; Esmaeilpour et al., 2021; von Conta et al., 2021; Violante et al., 2022). These simplified visualizations sometimes may bring misunderstanding of the underlying physics to the research community. For example, the graphical abstract of Grossman et al. (2017) is only a schematic that fails to illustrate the actual dynamics, which may lead one to believe that there is only one static focal spot between the two pairs of stimulation electrodes (Figure 1A). This can be frequently found in the IFS literature (Mirzakhalili et al., 2020; von Conta et al., 2021; Piao et al., 2022; Violante et al., 2022). See Figure 1 for a compilation of these visualizations of IFS. Although the electric field was modeled in these studies using state-of-the-art software packages, these schematic illustrations do not represent the complete dynamics. Here, we aimed to visualize the entire dynamic process of IFS including both the fast-oscillating carrier signals and the slowly oscillating modulation envelope, in the hope of contributing to the community with vivid educational resources on IFS as a powerful modality for non-invasive focal deep-brain stimulation.

## Materials and methods

### Construction of the head model

The forward head model was built on the ICBM152 (v6) template from the Montreal Neurological Institute (MNI, Montreal, Canada) (Mazziotta et al., 2001; Grabner et al., 2006), following the previously published methods (Huang et al., 2013). Briefly, the ICBM152 (v6) template magnetic resonance image (MRI) was segmented by the New Segment toolbox (Ashburner and Friston, 2005) in Statistical Parametric Mapping 8 (SPM8, Wellcome Trust Centre for Neuroimaging, London, UK) implemented in MATLAB (MathWorks, Natick, MA, USA). Segmentation errors such as discontinuities in the cerebrospinal fluid (CSF) and noisy voxels were corrected first by a customized Matlab script (Huang et al., 2013) and then by hand in interactive segmentation software Simpleware ScanIP (Simpleware Ltd., Exeter, UK). As TES modeling work has demonstrated the need to include the entire head down to the neck for realistic current flow, in particular in deep-brain areas and the brainstem (Huang et al., 2013), the field of view (FOV) of the ICBM152 (v6) MRI was extended down to the neck by registering and reslicing the standard head published in Huang et al. (2013) to the voxel space of ICBM152 (see Huang et al., 2016 for details). High-definition electrodes (6 mm radius) following the convention of the standard 10–10 international system (Klem et al., 1999) were placed on the scalp surface by a custom MATLAB script (Huang et al., 2013). Two rows of electrodes below the ears and four additional electrodes around the neck were also placed to allow for the targeting of deeper cortical areas and the use of distant reference electrodes in TES. A total of 93 electrodes were placed. A finite element model (FEM, Logan, 2007) was generated from the segmentation data by the ScanFE module in ScanIP. Laplace’s equation was then solved (Griffiths, 1999) in Abaqus 6.11 (SIMULIA, Providence, RI, USA) for the electric field distribution in the head. With one fixed reference electrode Iz as cathode, the electric field was solved for all other 92 electrodes with 1 mA current injected for each of them, giving 92 solutions for electric field distribution representing the forward model of the ICBM152 head.

### Optimization of the IFS montage

We employed previously published methods to optimize the montages for IFS. Specifically, we optimized the focality of modulation depth (MD) along the radial-in direction (Eq. 3 below) at the target with either two pairs of electrodes or two arrays of electrodes. Briefly, for the two pairs of electrodes, the optimization simply searches for the best two pairs that give the highest MD focality (Lee et al., 2020; Huang and Datta, 2021); for the two arrays of electrodes, the algorithm implements sequential quadratic programming to maximize the MD at the target while minimizing the energy of MD at the off-target areas (Huang et al., 2020). The target we picked is a deep location close to the left striatum with MNI coordinates of [−16, 10, 2] (Hampshire et al., 2019).

### Visualization of IFS dynamics

Suppose the optimized montages for the two stimulating currents are **s_1_**sin(ω_1_t) and **s_2_**sin(ω_2_t + π), where **s_1_** and **s_2_** are vectors of length 93 that encode the distribution of the current sources for each frequency ω_1_ and ω_2_, respectively. Here, we choose a phase difference of 180 degrees simply for visualization purposes. The total electric field in the brain induced by these two stimulating currents is


(1) E(r,t)=sin(ωt1)*A(r)s+1sin(ωt2+π)*A(r)s,2

where **A(r)** is the forward model of TES obtained above [also known as the lead field in the literature of EEG source localization (Dmochowski et al., 2017)]. **r** stands for any spatial location in the brain, and t is the time. The envelope of the interfering signal **E**(**r**, t) along a specific direction **d(r)** can be computed by the absolute value of the analytic signal:


(2) $$\left|{\text{E}}^{∼}\,\left(r,t\right)\right|=\left|\text{d}\,{\left(r\right)}^{T}\,\text{E}\,\left(r,t\right)+\mathrm{jH}\,\left[\text{d}\,{\left(r\right)}^{T}\,\text{E}\,\left(r,t\right)\right]\right|,$$

where j is the unit imaginary number, H[] is the Hilbert transform, and **d(r)** is a unit vector with | **d(r)**| = 1. The MD is defined as the depth of this envelope (Huang and Parra, 2019), i.e.,


MD(r)=max(|E(r,t)∼|)t-min(|E(r,t)∼|)t=

(3) 2min(|d(r)AT(r)s|1,|d(r)AT(r)s|2).

Note MD(**r**) is a static value that does not change with time and is the quantity we optimize (Huang et al., 2020; Huang and Datta, 2021). For visualization purposes, here we are interested in the instantaneous value of the MD, i.e.,


(4) MD(r,t)=|E(r,t)∼|-min(|E(r,t)∼|)t.

We also visualize the dynamics of the two stimulating currents, and the dynamics of the total electric field **E(r**, t) in 3D space as well as along a specific direction **d(r)***^T^***E(r**, t). We also visualize the distributions of **d(r)***^T^***E(r**, t) and MD(**r**, t) in a 2D brain slice. We visualize all these quantities for two specific directions **d(r)**: radial-in (pointing to the center of the brain, i.e., MNI coordinates of [0, 0, 0]) and posterior–anterior (PA, pointing to the front of the head), and at both the target location (left striatum) and a randomly chosen off-target location. We made animations to show the dynamics in action. For visualization purposes, we chose the two frequencies of the two stimulating currents to be only 10 Hz and 12 Hz and animated the dynamics for only 1 s.

## Results

### Visualization of electric field from two pairs of electrodes

**FIGURE 2:**
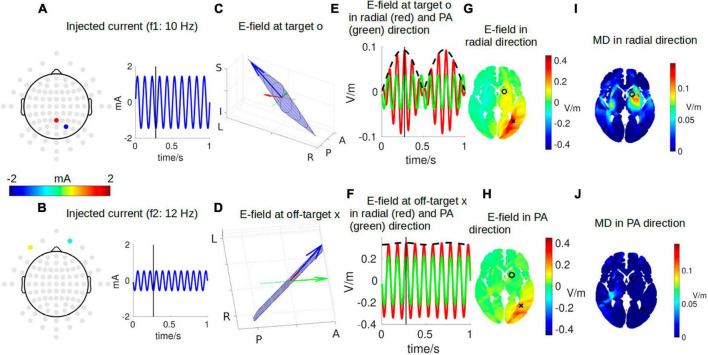
One instant in time during IFS stimulating the target location close to the left striatum with MNI coordinates of [–16, 10, 2] [indicated by a black circle in panel **(G)**]. **(A,B)** The optimal montages of two pairs of electrodes stimulating the target with the highest possible focality [as shown in panel **(I)**]. Note the topoplots in panels **(A,B)** show the amplitudes of the sinusoidal currents injected as shown by the right (gray circles are all candidate electrodes for the algorithm to consider when searching for the optimal montage). **(C,D)** The electric field (E-field) vector in 3D space (blue arrow). The head of the blue arrow moves in space and draws a Lissajous curve (black curve) in the 2D plane (blue plane) spanned by the two E-fields individually induced by the two stimulating currents. The orientation of the modeled head is indicated by letters on the three axes (L, left; R, right; P, posterior; A, anterior; I, inferior; S, superior). See Figure 3 for a zoomed-in version for more details. Specifically, we are interested in the E-field projected onto radial-in direction (red arrow) and posterior–anterior (PA) direction (green arrow). These three arrows are constantly moving, and panels **(C,D)** show the snapshots at the instant indicated by the black vertical lines in panels **(A,B,E,F)**. **(E)** E-field at the target along the radial direction (red) and PA direction (green). The black dashed line represents the envelope that defines the modulation depth (MD). **(F)** Same as **(E)** but for E-field at an off-target location indicated by a black cross in panel G. **(G,H)** Distribution of E-field along radial and PA directions in an axial slice through the target (black circle) and off-target (black cross) locations. **(I,J)** Distribution of MD along radial and PA directions in the same axial slice. Panels **(G,H,I,J)** are constantly changing, and shown here again are the snapshots at the instant indicated by the black vertical lines in panels **(A,B,E,F)**. For the complete animation, please see Supplementary Video 1.

Figure 2 shows a snapshot of the dynamical process of IFS at a time point of *t* = 0.272 s, indicated by the black vertical lines in panels A, B, E, and F. The optimal montage of two pairs of electrodes shown in panels A and B is determined by exhaustively searching through all the possible combinations from the 93 candidate electrodes (gray circles in panels A and B) (Lee et al., 2020; Huang and Datta, 2021). The optimal two pairs of electrodes are shown in Table 1. This optimal montage generates a maximal focal stimulation in terms of the MD as shown in panel I for the target location shown as a black circle.

**TABLE 1:** Optimal montages targeting the left striatum with MNI coordinates of [−16, 10, 2] using either two pairs of electrodes (Huang and Datta, 2021) or two arrays of electrodes (Huang et al., 2020).

	Frequency 1	Frequency 2
Two pairs of electrodes	Pz (1.45), PO4 (−1.45)	Ex15 (0.55), Ex18 (−0.55)
Two arrays of electrodes	T8 (0.357), Ex18 (0.170), F4 (0.169), FC6 (0.126), Fp2 (0.092), Ex10 (0.052), FC4 (0.030), PO4 (0.004), AF7 (−0.002), F3 (−0.002), Ex3 (−0.003), FT9 (−0.004), AF3 (−0.006), FC1 (−0.007), C2 (−0.008), Cz (−0.010), Nk2 (−0.012), C4 (−0.014), Exz (−0.024), P8 (−0.029), CP6 (−0.075), F8 (−0.804)	CP5 (0.875), TP8 (0.027), Fp2 (0.022), FT10 (0.018), AF4 (0.018), FC6 (0.016), F4 (0.011), T8 (0.007), C6 (0.002), AF8 (0.002), F6 (0.001), CP4 (−0.006), F1 (−0.020), CP2 (−0.059), O10 (−0.062), C2 (−0.080), P4 (−0.084), Fz (−0.097), AF3 (−0.115), Exz (−0.185), Ex11 (−0.291)

Numbers in the parentheses are the amplitudes (in mA) of the sinusoidal currents, with positive and negative values meaning currents going into and out of the head, respectively. Ex# electrodes are from the two additional rows of electrodes below the ears, and Nk2 is the electrode placed on the back of the neck (see Huang et al., 2013, for details).

**FIGURE 3:**
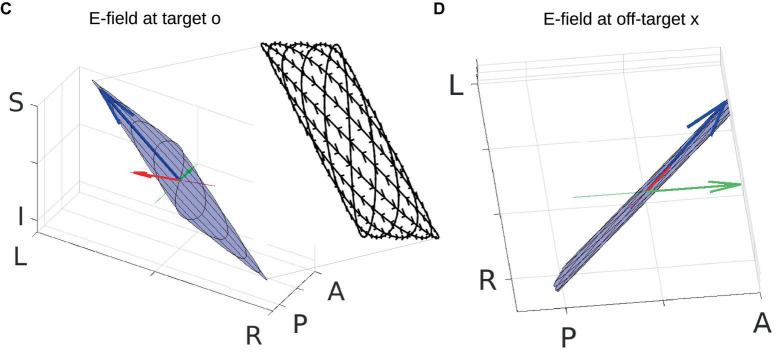
Zoom-in of panels **(C,D)** in Figure 2. Total electric field (E-field) is indicated by the blue arrow. Radial and posterior–anterior (PA) directions are indicated by the red and green lines, respectively, with projections of the E-field onto these two directions shown by red and green arrows. Blue planes represent the planes where the total E-field resides, and black curves are Lissajous curves drawn by the head of the moving blue arrows. The inset of panel **(C)** shows the Lissajous curves viewed in the PA direction (not drawn to scale), with small black arrows on the curves indicating the path of the head of the moving blue arrow. See Supplementary Video 1 for the entire dynamics. Orientation of the modeled head is indicated by letters on the three axes (L, left; R, right; P, posterior; A, anterior; I, inferior; S, superior).

The frequencies of the two stimulating currents are set as 10 Hz and 12 Hz for visualization purposes (Figures 2A, B). Each of these two currents induces an electric field (E-field) in the brain, and the two E-fields interfere with each other to generate a total E-field represented by the blue arrows in panels C and D. Due to the superposition of the two E-field vectors induced by the two stimulating currents, the total field always resides in the blue plane spanned by them, and the head of the total field traces a Lissajous curve in the blue plane. See Figure 3 for a zoomed-in version of the Lissajous curve, and the path the total field follows on that curve; also see Supplementary Video 1 for the complete animation. Unlike IFS, the conventional transcranial stimulation using alternating current generates an E-field that only oscillates along a 1D line, without any rotation of the field vector that traces a Lissajous curve in the 3D space.

Here, we are particularly interested in the projection of the total E-field along two exemplary directions: (1) the radial-in direction pointing to the center of the brain and (2) the posterior–anterior (PA) direction pointing toward the front of the head. These two directions are represented by the red and green lines, respectively, in Figures 2C, D and zoomed-in in Figure 3, where the projected E-fields are depicted by the red and green arrows. The alternating E-fields along these two directions are shown in Figures 2E, F, for the target and off-target locations shown in Figure 2G as a black circle and cross, respectively. The distributions of the instant E-field along radial and PA directions in an axial slice through the target location are shown in Figures 2G, H. The MD is the amplitude of the slowly oscillating envelope of the total E-field (e.g., black dashed line in Figure 2E). The distribution of the instant MD along radial and PA directions in the axial slice is in Figures 2I, J.

To summarize the relationship between different dynamics, the individual stimulating currents (Figures 2A, B) induce two fast-oscillating E-fields in the brain. These two E-fields interfere and generate a total E-field at the target location (blue arrow in Figure 2C). Projection of this E-field along the radial-in direction (red arrow in Figure 2C) traces the red waveform shown in Figure 2E, whose envelope (black dashed wave in Figure 2E) oscillates slowly and generates neuronal effects.

Note that the intensity of the instant E-field in the radial direction is weaker at the target than that at the off-target (0.09 vs. 0.34 V/m; Figure 2G), but the instant MD at the target is much higher than that at the off-target location (0.09 vs. 0.02 V/m; Figure 2I). This can also be seen from the red wave in Figure 2F whose envelope does not oscillate that much compared to that in Figure 2E. In fact, the MD is determined by the weaker of the two E-fields individually induced by the two stimulating currents (Eq. 3). At the off-target location, even though the total E-field is 0.34 V/m, the two stimulating currents individually induce an E-field of 0.33 V/m and 0.01 V/m. Therefore, the MD is very small. On the other hand, at the target location, the two stimulating currents individually induce an E-field of 0.04 V/m and 0.05 V/m, leading to a higher MD of 0.09 V/m (as seen in the black dashed line in Figure 2E) even though the instant E-field is smaller than that at the off-target location. We also note that we specifically optimized the MD along the radial direction (Figure 2I), and thus, the MD in the PA direction is weak for both the target and off-target locations (green waves in Figures 2E, F; also see Figure 2J). The animation (Supplementary Video 1) shows the entire dynamics. The E-field oscillates slowly in the animation as the frequencies of the system are only 10–12 Hz. In reality, the oscillation is much faster when the injected currents are in the 1 kHz range; that is, the carrier signal will oscillate at the kHz range, and the envelope oscillates at the ∼10 Hz range.

Note the MD in Figures 2I, J is usually illustrated as hotspots of stimulation right in the middle of the two pairs of electrodes in the literature (Figure 1). We found, however, that the hotspot (Figure 2I) does not exactly lie in the middle of the two pairs of electrodes, and there is more than one hotspot in the brain (e.g., the smaller hotspot in the left hemisphere in Figure 2I). In fact, the location of the hotspot cannot be intuitively determined from the electrode montage, and we employed numerical search to find the montage that gives the most focal MD at the predefined target. Also, the MD is sensitive to the specific direction as shown in Figures 2I, J.

### Visualization of electric field from two arrays of electrodes

**FIGURE 4:**
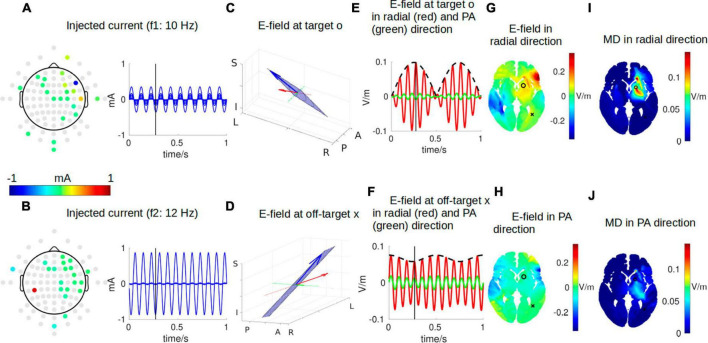
Same as Figure 2 but for two arrays of electrodes instead of two pairs of electrodes for focally stimulating the target. See Supplementary Video 2 for the entire animation.

A similar snapshot at the same time point of *t* = 0.272 s for two arrays of stimulating electrodes is shown in Figure 4. The array solutions are obtained using algorithms presented in Huang et al. (2020) to maximize the focality of MD along the radial-in direction at the target. The optimal two arrays of electrodes are shown in Table 1. When these montages are used, we achieve better focality of MD in radial-in direction at the target than that from using two pairs of electrodes. In fact, the smaller hotspot in the left hemisphere shown in Figure 2I disappears with array solutions shown in Figure 4I. Quantitatively, at a similar level of instant MD at the target (0.09 V/m), the focality of the MD is 3.72 cm from two arrays of electrodes (Figure 4I) and 4.93 cm from two pairs of electrodes (Figure 2I). Here, focality is defined as the cubic root of the volume in the brain that achieves over half of the MD at the target location (Huang and Parra, 2019), and thus, smaller number means higher focality.

## Discussion

This study attempts to give a complete visualization of the complicated dynamics of the underlying physics in IFS, including both the fast-oscillating carrier signals and the slowly oscillating modulation envelope as only static figures are available in the literature illustrating the core concept behind IFS. As the animation shows, the instant E-field at every single point in the brain oscillates and rotates fast in the 3D space. The premise of IFS is that neurons only respond to the slower oscillations of the envelope of these fast-changing E-fields, which is quantified by the modulation depth (MD) (Grossman et al., 2017; Huang and Parra, 2019). The hotspot of the MD does not exactly lie in the middle between the two pairs of stimulating electrodes as shown commonly in the literature. The animation shows how the MD is generated from the E-field and how it depends on the directions of interest. It also shows that the locations of the hotspot of MD cannot be intuitively determined, and more than one hotspot may present in the brain, with more focal hotspots if two arrays of electrodes are used. Note that the two directions we chose here (radial-in and posterior–anterior) are only exemplary for the purpose of visualization. The actual stimulation effects are highly correlated with the directions of the electric field relative to the cortical sheet (Rahman et al., 2013). However, the same physics applies to any direction of the electric field (Huang and Parra, 2019).

To the best of our knowledge, only a recent publication on IFS visualizes the complicated dynamics, but still in static figures (Wang et al., 2023). Here, we further show everything in action to give the readers a complete picture. We note that as the instant E-field rotates in the 3D space, it generates different strengths of MD along different directions. Existing optimization algorithms for IFS (Huang et al., 2020; Lee et al., 2020) only consider the spatial focality of the MD in a predefined direction, while ignoring the specificity of the modulation in different directions. In other words, it does not consider whether the optimal montage will also generate some strength of MD in directions other than the one being optimized that may modulate neurons in those directions. Future computational study will improve this by adding direction specificity to the cost function being optimized that only encodes spatial focality. In addition, multi-scale models that incorporate neuronal geometry are needed to investigate how the MD in different directions affects neurons at the target location (Wang et al., 2023). Finally, all these computational results of optimal IFS montages need to be validated by experimental recordings.

## Data availability statement

The original contributions presented in this study are included in this article/Supplementary material, further inquiries can be directed to the corresponding author.

## Author Contributions

The author confirms being the sole contributor of this work and has approved it for publication.

## Supplementary material

Supplementary Video 1 Full animation of the IFS dynamics stimulating the target location with two pairs of electrodes. Please refer to Figure 2 in the main manuscript for detailed explanation of each panel.

Supplementary Video 2 Full animation of the IFS dynamics stimulating the target location with two arrays of electrodes. Please refer to Figure 4 in the main manuscript for detailed explanation of each panel.
